# Monitoring of alien mosquitoes in Western Austria (Tyrol, Austria, 2018)

**DOI:** 10.1371/journal.pntd.0008433

**Published:** 2020-06-23

**Authors:** Hans-Peter Fuehrer, Ellen Schoener, Stefanie Weiler, Bita Shahi Barogh, Carina Zittra, Gernot Walder

**Affiliations:** 1 Department of Pathobiology, Institute of Parasitology, University of Veterinary Medicine Vienna, Vienna, Austria; 2 Dr. Gernot Walder GmbH, Austria; 3 Department of Limnology and Bio-Oceanography, University of Vienna, Vienna, Austria; 4 Division of Hygiene and Medical Microbiology, Medical University of Innsbruck, Innsbruck, Austria; INDEPENDENT RESEARCHER, UNITED STATES

## Abstract

Mosquitoes are of major importance to human and animal health due to their ability to transmit various pathogens. In Europe the role of mosquitoes in public health has increased with the introduction of alien *Aedes* mosquitoes such as the Asian tiger mosquito, *Aedes albopictus*; the Asian bush mosquito, *Ae*. *japonicus*; and *Ae*. *koreicus*. In Austria, *Ae*. *japonicus* has established populations in various regions of the country. *Aedes albopictus* is not known to overwinter in Austria, although isolated findings of eggs and adult female mosquitoes have been previously reported, especially in Tyrol. *Aedes koreicus* had not so far been found in Austria. Within the framework of an alien mosquito surveillance program in the Austrian province of Tyrol, ovitraps were set up weekly from May to October, 2018, at 67 sites– 17 in East Tyrol and 50 in North Tyrol. Sampling was performed at highways and at urban and rural areas. DNA obtained from mosquito eggs was barcoded using molecular techniques and sequences were analysed to species level. Eggs of alien *Aedes* species were found at 18 out of 67 sites (27%). Both *Ae*. *albopictus* and *Ae*. *japonicus* were documented at highways and urban areas in both East and North Tyrol. *Aedes koreicus* was found in East Tyrol. During this mosquito surveillance program, eggs of *Ae*. *albopictus*, *Ae*. *japonicus*, and *Ae*. *koreicus* were documented in the Austrian province of Tyrol. These findings not only show highways to be points of entry, but also point to possible establishment processes of *Ae*. *japonicus* in Tyrol. Moreover, *Ae*. *koreicus* was documented in Austria for the first time.

## Author summary

The importance of mosquitoes for Public Health in Europe increased dramatically with the introduction of alien species considered to be competent vectors of important human pathogens (e.g. dengue, chikungunya, and Zika viruses), which autochthonous mosquitoes are not yet known to transmit. The Asian tiger mosquito (*Aedes albopictus*), the Asian bush mosquito (*Aedes japonicus*), and *Aedes koreicus* are particularly of relevance, as they are expanding their range in Europe. Tyrol, a region in the Alps with main transport routes from Italy to Germany is of high relevance for the spread of potential invasive, alien *Aedes* mosquitoes. In this study, we demonstrate highways to be points of entry, and point to possible establishment of the Asian tiger mosquito and the East Asian bush mosquito in Tyrol (findings at highways and urban areas). Moreover, we report the first findings of *Ae*. *koreicus* in Austria, this species having most probably spread from neighbouring populations in Italy.

## Introduction

Blood-feeding mosquitoes play a major role in the transmission of pathogens. In Europe, their importance has increased in the past decade–mainly because of the introduction and establishment of invasive mosquitoes able to transmit pathogens which autochthonous mosquitoes are not able (or not known to be able) to transmit (e.g. chikungunya, dengue, and Zika viruses) [1]. Especially the Asian tiger mosquito, *Aedes* (*Stegomyia*) *albopictus* (Skuse, 1894), Asian bush mosquito, *Aedes* (*Hulecoeteomyia*) *japonicus* (Theobald, 1901), and *Aedes* (*Hulecoeteomyia*) *koreicus* (Edwards, 1917) have expanded their range in Europe in the past decade [2, 3]. A recent survey on alien species and human health in Austria lists invasive vectors such as *Ae*. *albopictus* as those alien species posing the most severe challenges [4].

The Asian tiger mosquito (*Ae*. *albopictus*) originates from subtropical and tropical Asian-Pacific regions and can actually be found on all continents with the exception of Antarctica [5]. The first report of Asian tiger mosquitoes in Europe originates from Albania in 1979 [6], but its European spread started from Italy (first discovery in 1990 in Genua or 1991 in Padova). Today, *Ae*. *albopictus* has been reported from more than 25 countries in Europe and established populations are known in at least 19 European countries [3]. Although overwintering and established populations are not known from Austria, this species has been reported to be introduced from Germany [7, 8], South Tyrol (northern Italy) [9], and other neighbouring regions. *Aedes albopictus* is of major public health concern, being a vector not only of various arboviruses such as dengue, chikungunya, Zika, and West Nile [5, 10], but also filarioid helminths [11]. Local outbreaks of chikungunya and dengue have been documented in Europe–two viruses not known to be transmitted by mosquitoes’ native in Europe (e.g. [12, 13]).

The native distribution area of the Asian bush mosquito, *Aedes japonicus*, is limited to temperate climatic regions of East Asia (China, Korea, Japan and south-eastern Russia). It is known as one of the most invasive mosquito species worldwide and has colonised 15 countries in Europe (e.g. Switzerland, Germany, Austria, France, Slovenia; summarized in [14, 15]) since its introduction. Under laboratory conditions *Ae*. *japonicus* is a competent vector of various pathogens: West Nile virus, Japanese encephalitis virus, chikungunya virus, dengue virus, *Dirofilaria repens*, and *D*. *immitis* (summarized in [14, 16, 17]). In field-sampled mosquitoes the Japanese encephalitis virus (in its native distribution range), but also West Nile Virus, La Crosse virus, and Cache Valley Virus (in the USA) have been detected (summarized in [14]). Recently the Usutu virus was documented in *Ae*. *japonicus* caught in the field in Graz, Austria [18].

*Aedes koreicus* is naturally distributed in East Asia from Japan, Korea, China, to parts of Eastern Russia. This invasive species was first documented in Europe in 2008 in a small area of 6 km^2^ in Belgium [19, 20]. In 2011, *Ae*. *koreicus* was found in the Veneto Region, North-Eastern Italy [21], where it spread rapidly [22–25]. Recently it was documented in the Northwest of Italy (Liguria; [26]). After the first findings in Italy, *Ae*. *koreicus* was also documented along the Swiss-Italian border, in Germany, Slovenia, and Hungary [27–32]. Under laboratory conditions, this mosquito is a competent vector for chikungunya virus and *Dirofilaria immitis* [33, 34]. *Dirofilaria repens* was documented in field-collected mosquitoes (however, its role as a potential vector is not known as its vector competence has not yet been determined) [35].

In the current study, alien mosquito species were monitored in the Western Austrian province of Tyrol. The study was conducted within the framework of the alien mosquito monitoring program of the federal state of Tyrol to evaluate the presence of alien *Aedes* spp. using ovitrapping at highways, but also in populated areas in Tyrol.

## Materials and methods

### Study area and sampling

Tyrol is a federal state in Western Austria and comprises the northern and eastern part of the historical princely county of Tyrol. North Tyrol borders on Germany (Bavaria) in the North, Vorarlberg in the West, Salzburg in the East, and Italy (South Tyrol) and Switzerland (Graubünden) in the South. East Tyrol shares its border with the Province of Belluno, Veneto region (Italy) and South Tyrol in the West. Potential larval sites (e.g. parking lots and petrol stations at highways, but also urbanized areas) were chosen as sampling sites. Ovitraps were set up weekly from May to October 2018 (calendar week 18–40) at 67 sites– 17 in East Tyrol and 50 in North Tyrol. They were installed in cities and villages (n = 53), at the Inn Valley Highway between Innsbruck and Kufstein (A12; n = 7), and the Brenner Highway between the Italian border and Innsbruck (A13; n = 7).

Ovitraps are widely used for surveillance of alien and invasive *Aedes* species as described elsewhere (e.g. [36]), and the same technique was used for the monitoring in 2017 [37]. Two conical black 500-ml cups filled with approximately 400 ml of water were set up per site. Wooden paddles were inserted as substrate for mosquito oviposition. Paddles were collected weekly and analysed for the presence of mosquito and other insect eggs under a dissection microscope. From each paddle *Aedes* eggs were pooled and transferred to 1.5-ml Eppendorf tubes for molecular analysis. Samples were stored at -20°C until further molecular analysis.

### Molecular mosquito specification

After homogenisation of eggs in a TissueLyser II (Qiagen, Germany) with two ceramic beads (2.8 mm Precellys Ceramic Beads, VWR, Germany) as described previously [37], DNA was isolated using the Qiagen DNeasy Blood&Tissue kit (Qiagen, Germany) according to the manufacturer’s instructions. To identify insect species, barcoding was performed within the mitochondrial cytochrome oxidase subunit I (mt COI) gene using the primers LepF1 and LepR1 [38]. PCR products were sequenced at LGC Genomics GmbH, Germany. Resulting sequences were compared to sequences available on BOLD Systems and GenBank databases. Aligned sequences were uploaded to GenBank (MN103383- MN103400).

## Results

Eggs of alien *Aedes* species were found at 18 of 67 Tyrolean sites (27%). (Potentially) invasive mosquito species were more common at highways (7/14; 50%) than in other areas (11/53; 21%; Table 1). At the Inn Valley Highway A12 (5/7; 71%) more sites were positive for mosquito eggs than at the Brenner Highway A13 (2/7; 29%). In East Tyrol at 41% of the sites (7/17) eggs of alien mosquitoes were documented, whereas in North Tyrol 8% (4/50; only in urban areas–Innsbruck and Kufstein) gave positive findings (Table 2). Given the relatively small sample sizes, these differences were not statistically significant.

**Table 1:** Eggs of alien mosquito species collected in positive individual ovitraps in 2018 along highways (Inn Valley Highway A12 and Brenner Highway A13) in Tyrol, Austria (nd = sequences were not uploaded to GenBank e.g., short sequences or poor sequence quality).

Location/ID	Highway	Longitude	Latitude	Date	Species found	Number of *Aedes* spp. eggs present	GenBank ID
Brenner (petrol station)/1	A13	47.006908	11.509468	Sep. 19	*Ae*. *japonicus*	1	nd
Brenner (petrol station 2)/2	A13	47.039508	11.473567	Aug. 15	*Ae*. *japonicus*	41	MN103383
Parking Site Volders/3	A12	47.282405	11.552238	July 26	*Ae*. *japonicus*	28	MN103385
				Aug. 8	*Ae*. *albopictus*	30	MN103394
				Aug. 29	*Ae*. *albopictus*	112	MN103394
				Sep. 5	*Ae*. *japonicus*	19	MN103385
Rest stop Weer South/4	A12	47.317873	11.655833	Aug. 8	*Ae*. *albopictus*	45	MN103393
				Sep. 26	*Ae*. *albopictus*	5	MN103394
Parking Site Münster South/5	A12	47.410918	11.842285	July 5	*Ae*. *japonicus*	6	MN103385
				Aug. 1	*Ae*. *albopictus*	49	MN103394
				Aug. 8	*Ae*. *albopictus*	7	MN103394
				Sep. 5	*Ae*. *albopictus*	1	nd
Truck Control Station/6	A12	47.457978	11.920342	Aug. 29	*Ae*. *japonicus*	19	MN103390
Parking Site Langkampfen/7	A12	47.541167	12.114102	June 28	*Ae*. *japonicus*	57	MN103383

**Table 2:** Eggs of alien mosquito species collected in villages and cities in Tyrol, Austria (nd = sequences were not uploaded to GenBank e.g., short sequences or poor sequence quality).

Location	Longitude	Latitude	Date	Species	Number of *Aedes* spp. eggs present	Corine Land Cover (Level 3)	GenBank ID
**East Tyrol**							
Lienz–cemetery St. Andrä/8	46.834270	12.759662	July 11	*Ae*. *japonicus*	13	Discontinuous urban fabric	MN103384
Lienz—red cross/9	46.834190	12.766865	July 4	*Ae*. *japonicus*	62	Discontinuous urban fabric	MN103384
			July 25	*Ae*. *japonicus*	48		MN103386
			Aug. 9	*Ae*. *japonicus*	141		nd
			Aug. 16	*Ae*. *koreicus*	22		MN103399
Lienz–Fischwirtbrücke/10	46.831458	12.769565	July 25	*Ae*. *japonicus*	20	Continuous urban fabric	MN103387
Lienz–Tiroler Strasse/11	46.827560	12.767468	July 18	*Ae*. *japonicus*	51	Continuous urban fabric	MN103384
Lienz–near fire brigade/12	46.826782	12.759982	July 11	*Ae*. *albopictus*	12	Discontinuous urban fabric	MN103394
			July 18	*Ae*. *albopictus*	137		nd
Tristach/13	46.814563	12.805695	Sep. 20	*Ae*. *japonicus*	54	Non-irrigated arable land	MN103389
Nörsacher Teiche/14	46.766280	12.931955	Sep. 27	*Ae*. *japonicus*	187	Land principally occupied by agriculture, with significant areas of natural vegetation	nd
**North Tyrol**							
Innsbruck–Badhaus/15	47.281683	11.406097	Aug. 8	*Ae*. *albopictus*	3	Discontinuous urban fabric	MN103394
Kufstein–Site 1/16	47.556250	12.118567	July 26	*Ae*. *japonicus*	13	Industrial or commercial units	MN103388
			Aug. 8	*Ae*. *japonicus*	86		nd
			Aug. 29	*Ae*. *japonicus*	34		MN103391
Kufstein–Airport/17	47.566167	12.126500	June 14	*Ae*. *albopictus*	11	Discontinuous urban fabric	MN103394
			July 12	*Ae*. *japonicus*	7		nd
			Aug. 29	*Ae*. *japonicus*	57		MN103391
			Sep. 26	*Ae*. *japonicus*	66		MN103392
Kufstein–Site 2/18	47.565967	12.145733	June 21	*Ae*. *japonicus*	119	Discontinuous urban fabric	MN103383
			Sep. 5	*Ae*. *japonicus*	151		MN103385

**Fig 1:**
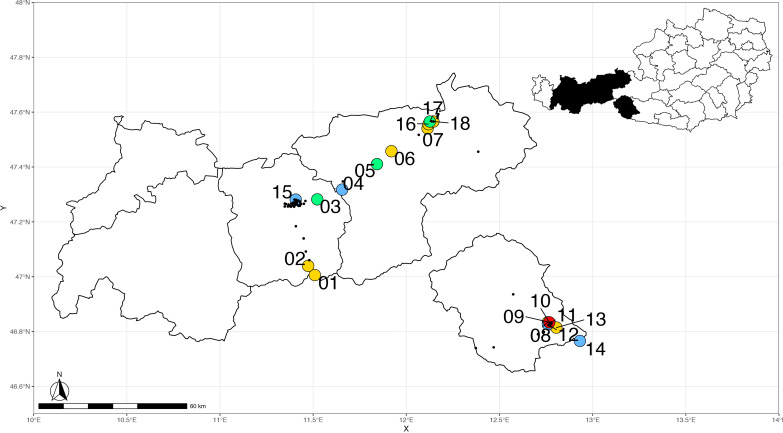
Distribution of *Aedes albopictus*, *Ae*. *japonicus* and *Ae*. *koreicus* (2018) in Tyrol (2018). Black dots: negative sites, red dot: *Ae*. *koreicus* and *Ae*. *japonicus*, yellow dot: *Ae*. *albopictus*, blue dot: *Ae*. *japonicus*, green dots: *Ae*. *albopictus* and *Ae*. *japonicus*. Numbers indicate the sampling site as given in Tables 1 and 2.

*Aedes albopictus* was documented at six of 67 sites (9%; Fig 1). In total, three out of 14 sites at highways were positive (21%). However, in this study tiger mosquitoes were only found at the Inn Valley Highway (3/7; 43%). No *Ae*. *albopictus* eggs were detected at the Brenner Highway. However, eggs were detected in urban areas in East Tyrol (1/17; 6%) in Lienz and in North Tyrol (2/36; 6%) in Innsbruck and Kufstein. Sequence analysis revealed two haplotypes (100% identity to MH817558 and KC690954).

*Aedes japonicus* was documented at 15 of 67 sites (22%; Fig 1) both at highways (6/14; 43%) and other areas (9/53; 17%). This species was detected at the Brenner Highway (2/7; 29%) close to the Italian border to South Tyrol, and the Inn Valley Highway (4/7; 57%). In East Tyrol, it was found in rural and urban areas (6/17; 35%). In North Tyrol (excluding highways) eggs of *Ae*. *japonicus* were detected at three of 50 sampling sites (6%) all located in Kufstein. Genetic analysis revealed 10 haplotypes of *Ae*. *japonicus* within the mt COI barcode region (see Tables 1 and 2).

*Aedes koreicus* eggs were found at a single site in Lienz, East Tyrol, in August 2018 (Fig 1). At sequence analysis, the sample was 100% identical to *Ae*. *koreicus* KM258298 collected in Maasmechelen, Belgium [39].

Additionally, eggs of autochthonous mosquito species were documented in this study, namely *Aedes geniculatus* (July and September 2018), *Aedes* sp. (September 2018; MN103396), *Anopheles plumbeus* (October 2018), *Culex pipiens* complex (July 2018), and *Culex torrentium* (June and August 2018; MN103395). The following dipteran non-mosquito insect eggs were documented: 0.4–0.5 mm long brownish eggs of *Clogmia albipunctata* (June 2018; Psychodidae; MN103400), 0.6 mm brownish eggs of *Sphegina clunipes* (July 2018; Syrphidae; MN103398), and 0.3 mm long white eggs of Chloropidae (August 2018; grass flies; MN103397). At sequence analysis of pooled samples, double-peaks indicating numerous different *Aedes* species were observed at one sample at Kufstein-Airport (September 18^th^ 2018). However, it might have failed at other pooled samples.

## Discussion

The first report of *Ae*. *albopictus* in Austria was based on findings of immature stages and a single female mosquito in 2012 in Jennersdorf, Burgenland, and *Ae*. *albopictus* larvae in Angath, Tyrol, approximately 2 km from the Inn Valley Highway [40]. Reports of single female mosquitoes and eggs were subsequently documented along the Inn Valley Highway, which prompted a continuous monitoring of this area using ovitraps. Our findings of *Ae*. *albopictus* eggs along the Inn Valley Highway were expected. In 2017, eggs of tiger mosquitoes were documented in two of the five positive sites from 2018 (Rest stop Weer South and Parking Site Münster South; [37]). In Bavaria (e.g. Kiefersfelden close to the Austrian border and Kufstein), *Ae*. *albopictus* was also found at service stations, associated with transit road traffic [41]. The dispersal of adult *Ae*. *albopictus* by car has recently been shown in Spain [42].

In 2017, in East Tyrol, a single *Ae*. *albopictus* egg was reported from an ovitrap set up in Tassenbach. This sampling site was negative for tiger mosquitoes in 2018, but *Ae*. *albopictus* was found in Lienz on July 11^th^ and 18^th^ 2018. In North Tyrol, *Ae*. *albopictus* was found in Kufstein in 2017 [37] and in Innsbruck and Kufstein in 2018. These findings indicate not only introduction but also a possible establishing process, but further studies are needed to prove this hypothesis. Overwintering populations and possible establishment of tiger mosquitoes has been reported from neighbouring Italy (e.g. South Tyrol and Trento; e.g. [43]) and Germany (e.g. Freiburg; [44, 45]) but so far not from Tyrol or other parts of Austria.

The first findings of *Ae*. *japonicus* in Austria were reported in 2011. Larvae of the Asian bush mosquito were found in a hand basin in the area of Kreuzberg, Southern Styria, in South Eastern Austria (close to Slovenia; [40]). By 2015, *Ae*. *japonicus* had expanded its range eastwards into Burgenland and westwards to Carinthia [46, 47], and by 2018, further to Friuli Venezia Giulia and Veneto Region in North-Eastern Italy [48]. Between 2014 and 2017, a spread from southern Burgenland northwards to Lower Austria and the capital city of Vienna was observed [37, 49]. In the most western province of Austria, Vorarlberg, populations of *Ae*. *japonicus* were documented in 2015 (possibly originating from Switzerland; [47]). In Salzburg, these mosquitoes were found in 2015 (possibly originating from South-Eastern Austria; [50]) and in Upper Austria in 2018 [18].

The only Austrian province (almost) free (exception Kufstein area) of these mosquito species was Tyrol with the “Swiss/Vorarlberg” *Ae*. *japonicus* population in the West, the “Carinthian/Italian” population in the South and the “Salzburg/Upper Bavarian” population in the East. In 2017, no *Ae*. *japonicus* eggs were reported [37]. By contrast, 15 of 67 sampling sites were positive for *Ae*. *japonicus* eggs in 2018. *Aedes japonicus* was documented at two petrol stations at the Brenner Highway close to the Italian border and at the Inn Valley Highway, indicating vehicular traffic as a route of introduction. However, this mosquito species might also have reached this area by active flight. Moreover, *Ae*. *japonicus* was found in East Tyrol (e.g. Lienz), indicating dispersal to this area from Italy in the South or Carinthia in the East. The findings of *Ae*. *japonicus* in Kufstein were associated with populations in Upper Bavaria/Germany. According to Koban et al. [51], four populations of Asian bush mosquitoes are currently present in Central Europe. This study indicates that the two biggest populations (Western-German/Swiss/French and Southern-Austrian/Slovenian/Italian) might encounter each other in North Tyrol. Population genetic studies (nad4 mitochondrial locus and microsatellite analysis [52]) might resolve this question, but a recent study demonstrated that the origin of entry into Germany cannot be clarified ten years after the first detection in that country [14]. Active spread, re-introduction, and carry-overs may take place regularly [14].

Although *Ae*. *koreicus* was reported in North-East Italy and Slovenia, this mosquito species had not been reported from Austria until now. In the present study, *Ae*. *koreicus* eggs were found for the first time in August 2018 in Lienz, East Tyrol. The Italian region Veneto (where *Ae*. *koreicus* is known to be present) borders onto East Tyrol [48]. However, with the findings of eggs in ovitraps at only one location, it remains unclear if this mosquito has spread to Austria or if it was an isolated introduction. Establishment in hilly and pre-alpine areas up to an altitude of 800 m in Italy [21, 22] indicates that establishment and further distribution can be expected in Austria in the coming years.

Fifty culicid species (Diptera: Culicidae; genera: *Aedes*, *Anopheles*, *Culex*, *Coquillettidia*, *Culiseta*, *Ochlerotatus*, *Orthopodomyia*, and *Uranotaenia*) have been detected in Austria so far [53]. With the finding of *Ae*. *koreicus* this number increases to 51 species.

This study has some limitations. Barcode primers (mt COI) known to bind these mosquito species were used. Analysis of pooled mosquito eggs might have meant that mosquito species were overlooked if more than one mosquito species laid eggs on a paddle, but this technique had been chosen because until this monitoring program only *Ae*. *albopictus* was reported in the studied area (37).

We focused on alien *Aedes* mosquitoes and therefore used ovitraps only. This technique is cheap, easy to use, and an effective tool for monitoring alien mosquito species. Further research (e.g. inclusion of adult mosquito sampling) and surveillance is needed to evaluate overwintering, establishment, and invasive behaviour of *Ae*. *albopictus*, *Ae*. *japonicus*, and *Ae*. *koreicus* in the Austrian province of Tyrol, but also in other regions in Austria.

Using ovitraps, *Ae*. *albopictus*, *Ae*. *japonicus*, and *Ae*. *koreicus* were found in the Austrian province of Tyrol. *Aedes albopictus* and *Ae*. *japonicus* were both documented not only at highways but also in urban areas in Tyrol. These findings not only demonstrate that highways are points of entry but also point to possible establishment processes in Tyrol. To the best of our knowledge this is the first report of the presence of *Ae*. *koreicus* in Austria. The risk of the establishment of especially *Ae*. *albopictus* (but also *Ae*. *koreicus* and *Ae*. *japonicus*) in North and East Tyrol is clear, and informing the public and stakeholders about measures to hamper this development is highly recommended.
